# Rotavirus-Specific Immunoglobulin A Responses Are Impaired and Serve as a Suboptimal Correlate of Protection Among Infants in Bangladesh

**DOI:** 10.1093/cid/ciy076

**Published:** 2018-01-31

**Authors:** Benjamin Lee, Marya Carmolli, Dorothy M Dickson, E Ross Colgate, Sean A Diehl, Muhammad Ikhtear Uddin, Shahidul Islam, Motaher Hossain, Tanzeem Ahmed Rafique, Taufiqur Rahman Bhuiyan, Masud Alam, Uma Nayak, Josyf C Mychaleckyj, Monica M McNeal, William A Petri, Firdausi Qadri, Rashidul Haque, Beth D Kirkpatrick

**Affiliations:** 1 Department of Pediatrics, Vaccine Testing Center, University of Vermont Larner College of Medicine, Burlington; 2 Department of Medicine, Vaccine Testing Center, University of Vermont Larner College of Medicine, Burlington; 3 International Centre for Diarrhoeal Disease Research, Dhaka, Bangladesh; 4 Center for Public Health Genomics and Department of Public Health Sciences, University of Virginia, Charlottesville; 5 Laboratory of Specialized Clinical Studies, Cincinnati Children’s Hospital Medical Center, Ohio; 6 Division of Infectious Diseases and International Health, University of Virginia, Charlottesville

**Keywords:** rotavirus, IgA, vaccination, correlate of protection, Prentice criteria

## Abstract

**Background:**

Rotavirus (RV)–specific immunoglobulin A (IgA) responses following oral RV vaccination are impaired in low-income countries, where the utility of RV-IgA as a correlate of protection (CoP) remains unclear. In a monovalent oral RV vaccine (Rotarix) efficacy trial among infants in Dhaka, Bangladesh, we identified factors associated with poor RV-IgA responses and explored the utility of RV-IgA as a CoP.

**Methods:**

Infants were randomized to receive Rotarix or no Rotarix at 10 and 17 weeks of life and followed with active diarrheal surveillance. RV-IgA concentration, seroconversion, and seropositivity were determined at 18 weeks of life and analyzed for correlation(s) with rotavirus diarrhea (RVD) and for contribution to Rotarix vaccine effect.

**Results:**

Among vaccinated infants, overall RV-IgA geometric mean concentration was 21 U/mL; only 27% seroconverted and 32% were seropositive after vaccination. Increased RV-specific maternal antibodies significantly impaired immunogenicity. Seroconversion was associated with reduced risk of RVD through 1 year of life, but RV-IgA seropositivity only explained 7.8% of the vaccine effect demonstrated by the clinical endpoint (RVD).

**Conclusions:**

RV-IgA responses were low among infants in Bangladesh and were significantly impaired by maternal antibodies. RV-IgA is a suboptimal CoP in this setting; an improved CoP for RV in low-income countries is needed.

**Clinical Trials Registration:**

NCT01375647.

## Teaser

Monovalent oral rotavirus vaccine was poorly immunogenic among infants in Bangladesh. The standard measure of vaccine immunogenicity, rotavirus-specific immunoglobulin A, appeared to be a suboptimal correlate of protection (CoP) in this population, suggesting that improved CoPs are needed.

Rotavirus (RV) remains the leading cause of diarrhea among infants worldwide [1, 2]. Oral, live attenuated RV vaccines have significantly reduced RV disease, with worldwide deaths due to RV among children having decreased from 528000 in 2000 to 215000 in 2013 [3]. However, vaccines are only half as effective in low-income countries (LICs), where child mortality is high and disease burden is greatest, compared with high-income countries [3–5]. Efforts to close this gap in vaccine efficacy (VE) are hindered by incomplete understanding of the factors mediating vaccine immunogenicity and lack of a reliable correlate of protection (CoP) for RV.

The standard measure of RV vaccine immunogenicity is serum RV-specific immunoglobulin A (RV-IgA), which correlates with VE at the population level, especially in high-income countries [6]. Factors proposed to impact RV-IgA responses include maternally derived antibodies, breast milk antibodies, micronutrient deficiencies, and enteric coinfections and enteropathy [7]. Effective interventions to address these factors remain elusive, indicating the need for improved understanding of their contributions to vaccine immunogenicity.

A separate matter is whether RV-IgA can serve as a CoP for RV and predict protection from RV diarrhea (RVD) at the individual level, particularly in LICs. A CoP would greatly accelerate vaccine research by obviating the need for clinical endpoints (ie, RVD) in future trials. RV-IgA has been proposed as the best candidate [6, 8]. However, few studies have investigated RV-IgA as a CoP specifically in LICs, where a CoP is most needed [9].

Improved knowledge of RV-IgA responses at both the population and individual level is thus mandatory to maximize reductions in the global burden of RV disease. Therefore, in a randomized trial of monovalent oral RV vaccine (Rotarix) VE performed in urban Dhaka, Bangladesh, we identified factors that significantly impacted vaccine immunogenicity, determined the correlation between RV-IgA and protection from RVD at the individual level, and evaluated RV-IgA as a CoP using Prentice criteria [10].

## METHODS

### Study Design

The Performance of Oral Vaccines in Developing Countries (PROVIDE) study was a birth cohort study performed from 2011 to 2014 in urban Dhaka, Bangladesh, that included a randomized controlled trial to evaluate the efficacy of a delayed Rotarix schedule. Study design, procedures, and primary results have been reported previously [11–15]. In brief, 700 infants were randomized 1:1 to receive Rotarix or no Rotarix at 10 and 17 weeks of life and followed by active diarrheal surveillance. Standard vaccines were administered according the Expanded Programme on Immunization (EPI) schedule. The study was approved by the ethical review boards of the International Centre for Diarrhoeal Disease Research, Bangladesh, the University of Vermont, and the University of Virginia, and was registered at ClinicalTrials.gov (NCT01375647).

### Procedures

Each diarrheal episode (≥3 abnormally loose stools within 24 hours) was tested for RV using the ProSpecT enzyme immunoassay (EIA) kit (Oxoid Ltd, Hampshire, United Kingdom). Plasma was collected at 6 and 18 weeks (before and 1 week after vaccination) in all infants and at week 24 in a subset. Breast milk was collected prior to week 6. Plasma RV-IgA, plasma RV-specific immunoglobulin G (RV-IgG), and breast milk RV-IgA were measured by EIA: 96-well microtiter plates were coated with anti-RV rabbit hyperimmune serum raised against a pool of RVs (strains SA-11, RV3, RV4, RV5, and ST3), and simian SA11-strain RV added as antigen, as previously described [16]. IgA/IgG was detected using peroxidase-conjugated secondary antibody followed by tetramethylbenzidine reaction to measure antibody concentration (U/mL) (Supplementary Materials). Values ≤7.5 U/mL (the lower limit of detection) were assigned 7.5 U/mL. Seropositivity was defined as RV-IgA ≥20 U/mL. Seroconversion was defined as RV-IgA ≥20 U/mL with week 6 RV-IgA <20 U/mL.

### Statistical Analysis

Statistical analysis was performed using SPSS version 24 (IBM, Armonk, New York) and GraphPad Prism version 7.01 (GraphPad Software, La Jolla, California) software. Geometric mean and log geometric mean antibody concentrations (GMCs) were calculated for the year 1 per-protocol population. Mann-Whitney *U* test was used to compare RV-IgA concentration between groups. The χ^2^ or Fisher exact test was used to compare differences in categorical outcomes. Logistic regression was performed to determine associations between RV-IgA seroconversion and RVD through 1 year of age, to examine factors associated with RV-IgA seropositivity, and to test for interactions among variables. A multivariable model was created that included all factors significant in univariate analysis at *P* < .125. Using an outcome of any RVD from week 18 to week 52 (1 week postvaccination through year 1), the proportion of vaccine effect explained by RV-IgA was calculated as 1 minus the ratio of the logistic model coefficients obtained from a model with vaccine only, and 1 with vaccine plus RV-IgA [17]. Statistical significance was set at a 2-sided *P* value <.05.

## RESULTS

### RV-IgA Responses Were Poor Among Infants in Bangladesh

**Figure 1.**
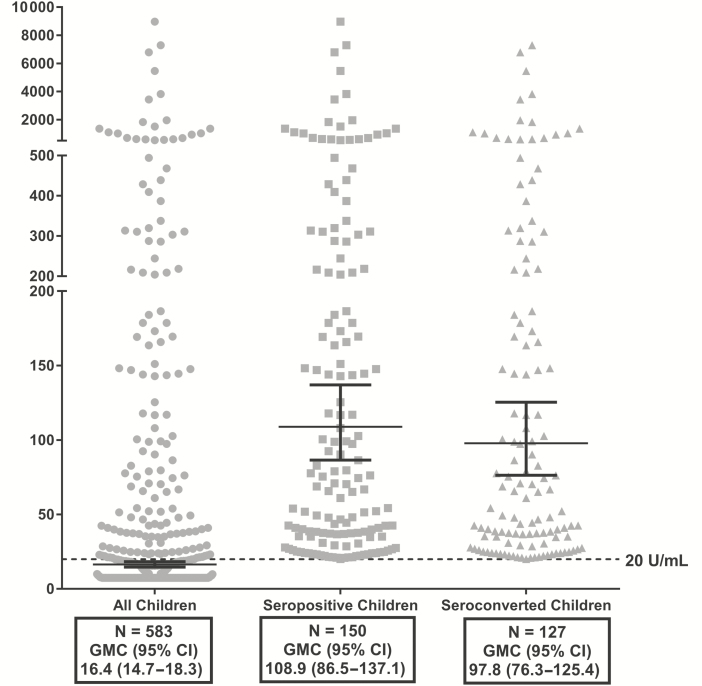
Rotavirus-specific immunoglobulin A (RV-IgA) geometric mean concentration (U/mL) among all, seropositive, and seroconverted children at week 18 of life. Dashed line represents 20 U/mL, the threshold for seropositivity. Seroconversion was defined as week 18 RV-IgA ≥20 U/mL with week 6 RV-IgA <20 U/mL. Abbreviations: CI, confidence interval; GMC, geometric mean concentration.

Among 583 children with week 18 RV-IgA results, the RV-IgA GMC was 16.4 U/mL (95% confidence interval [CI], 14.7–18.3 U/mL; Figure 1). The RV-IgA GMC among seropositive children was 108.9 U/mL (95% CI, 86.5–137.1 U/mL), but only 26% (n = 150) were seropositive (Figure 1). Five hundred seventy-five children with both week 6 and week 18 RV-IgA measurements were included for seroconversion analysis. The RV-IgA GMC among seroconverters was 97.8 U/mL (95% CI, 76.3–125.4 U/mL), but only 22% seroconverted (n = 127; Figure 1).

Among infants included in the seropositivity analysis, 49.5% (n = 289) were vaccinated, of whom 32% (n = 93) were seropositive postvaccination. The week 18 RV-IgA GMC was higher in vaccinated infants (21.0 U/mL [95% CI, 17.6–24.9 U/mL]) than in unvaccinated infants (12.9 U/mL [95% CI, 11.3–14.7 U/mL; *P* < .001; Table 1). Among children with both week 6 and week 18 RV-IgA results included for seroconversion analysis, 49.4% (n = 284) were vaccinated (Table 1). Only 27% (n = 77) of vaccinated infants seroconverted (Table 1). No significant differences in GMC were observed in vaccinated compared to unvaccinated children. One hundred seventy-seven children had week 24 RV-IgA results: 85 were vaccinated (49.7%), of whom 36 (42.4%) were seropositive and 33 (38.8%) had seroconverted (Table 1). Among vaccinated infants with week 24 seroconversion, 16 (48%) had newly seroconverted since week 18. Among vaccinated infants negative for week 24 seroconversion, 8 (15%) had seroconverted at week 18 but subsequently seroreverted. In infants with paired week 18 and week 24 specimens, no significant differences were seen in RV-IgA concentration.

**Table 1.** Rotavirus-Specific Plasma Immunoglobulin A Geometric Mean Concentration (U/mL) at Weeks 6 and 18 of Life

Group	Week 6				Week 18				Week 24			
	Vaccinated		Unvaccinated		Vaccinated		Unvaccinated		Vaccinated		Unvaccinated	
	No.	GMC (95% CI)	No.	GMC (95% CI)	No.	GMC (95% CI)	No.	GMC (95% CI)	No.	GMC (95% CI)	No.	GMC (95% CI)
All	284	8.7 (8.2–9.2)	291	8.5 (8.0–9.1)	289^a^	21.0 (17.6–24.9)^b^	294^a^	12.9 (11.3–14.7)^b^	85^a^	27.6 (19.2–39.7)^c^	92^a^	20.7 (14.8–28.8)^c^
Seropositive	…	…	…	…	93	120.5 (88.3–164.4)	57	92.4 (65.8–129.7)	36	133.7 (80.5–222.1)	30	147.8 (87.2–250.6)
Seroconverted	…	…	…	…	77	106.1 (75.8–148.4)	50	86.4 (59.6–125.3)	33	139.5 (81–240.2)	27	150.0 (84.4–266.6)

Abbreviations: CI, confidence interval; GMC, geometric mean concentration.

^a^Includes infants without week 6 measurements.

^b^ *P* < .001.

^c^ *P* = .081.

A subset of infants (n = 317) had sufficient plasma for RV-IgG measurement at weeks 6 and 18. In this subset, RV-IgG GMC was significantly lower at week 18 (78.4 U/mL [95% CI, 69.7–88.2 U/mL]) than week 6 (292.7 U/mL [95% CI, 254.2–337.0 U/mL]; *P* < .0001), reflecting waning of maternal antibodies. Week 18 RV-IgG concentration did not differ between vaccinated (52%) and unvaccinated infants (data not shown).

The standard EIA for RV-IgA developed by Ward and colleagues at Cincinnati Children’s Hospital uses human-strain 89-12 RV as antigen [18]. To evaluate for possible differences in the PROVIDE EIA, 40 specimens were measured at Cincinnati. RV-IgA concentrations were consistently lower as determined by PROVIDE, but most (87.5%) were concordant for serostatus (Supplementary Table 1).

### Vaccination and Maternally Derived Factors Are Associated With RV-IgA Responses

**Figure 2.**
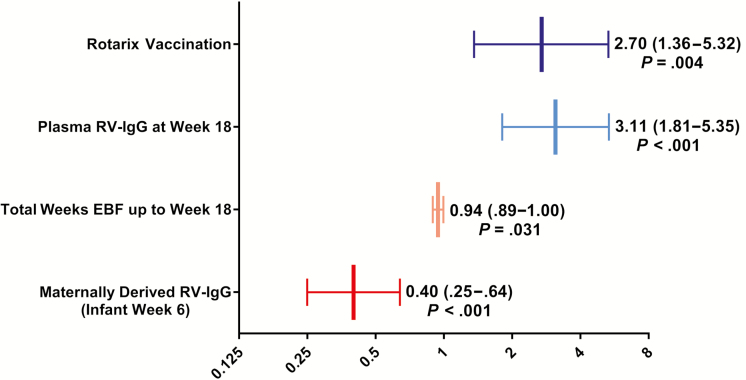
Factors significantly associated with rotavirus-specific immunoglobulin A seropositivity. Values shown represent adjusted odds ratios (x-axis) with corresponding 95% confidence intervals in a multivariable model that included 10 variables significant in univariate analysis at *P* < .125. Abbreviations: EBF, exclusive breastfeeding; RV-IgG, rotavirus-specific serum immunoglobulin G.

Univariate analyses were performed to assess factors associated with RV-IgA response [12, 13]. Ten variables were ultimately included in a multivariable model performed in children with complete data (n = 235): Rotarix, week 6 and week 18 RV-IgG, water treatment, shared toilet, enrollment height-for-age *Z* score, week 6 and week 18 plasma vitamin D, weeks of exclusive breastfeeding (EBF) until week 18, and breast milk RV-IgA. Rotarix (odds ratio [OR], 2.7 [95% CI, 1.36–5.32]) and week 18 RV-IgG (OR, 3.11 [95% CI, 1.81–5.35]), which would reflect RV-IgG induced by vaccination or natural infection, were positively associated with RV-IgA seropositivity (Figure 2); week 6 RV-IgG (ie, maternal antibodies) had a strong inverse correlation with seropositivity (OR, 0.40 [95% CI, .25–.64]), and EBF was also associated with reduced seropositivity (OR, 0.94 [95% CI, .89–1.0]; Figure 2). No significant interactions were detected between vaccination and any other variable.

We further characterized the effect of maternally derived RV-IgG in Rotarix recipients (n = 153). All vaccinated infants with week 6 RV-IgG above the 90th percentile (1331 U/mL) failed to seroconvert (*P* = .007); vaccinated infants above the 75th percentile (703 U/mL) were less likely to seroconvert (Table 2). In those who failed to seroconvert, week 6 RV-IgG GMC (329 U/mL [95% CI, 253–427 U/mL]) was significantly higher than among seroconverters (171 U/mL [95% CI, 115–254 U/mL]; Table 2). Similar results were observed for seropositivity (data not shown).

**Table 2.** Week 6 Rotavirus-Specific Plasma Immunoglobulin G and Immunoglobulin A Seroconversion Among Vaccinated Infants

Week 6 RV-IgG	RV-IgA Seroconversion		*P* Value
Cutoff, U/mL	Yes	No	
<1331	43	95	
≥1331^a^	0	15	.007
<703	37	78	
≥703^b^	6	32	.051
GMC, U/mL (95% CI)	171 (115–254)	329 (253–427)	.012

Abbreviations: CI, confidence interval; GMC, geometric mean concentration; RV-IgA, rotavirus-specific immunoglobulin A; RV-IgG, rotavirus-specific immunoglobulin G.

^a^Ninetieth percentile, week 6 RV-IgG among vaccinated infants.

^b^Seventy-fifth percentile, week 6 RV-IgG among vaccinated infants.

### RV-IgA Is Associated With RVD but Does Not Fully Predict Protection

We then evaluated whether RV-IgA seroconversion was associated with RVD in infants with complete 1 year of follow-up. Seroconverters had a reduced risk of RVD (relative risk, 0.52 [95% CI, .34–.81]), and seroconverted infants without RVD had higher GMC compared to those with RVD; these effects were observed in both vaccinated and unvaccinated infants (Table 3). Seroconversion remained associated with reduced RVD risk after adjusting for RVD before week 18 and for vaccine arm (*P* = .024). Although seroconversion was associated with reduced RVD risk, protection was incomplete. Among children with RVD, 15% (n = 19) had seroconverted, whereas 74.4% (n = 311) who were RVD-free had failed to seroconvert.

**Table 3.** Rotavirus-Specific Immunoglobulin A Seroconversion and Protection From Rotavirus Diarrhea Through 1 Year of Life

Group	Seroconversion	No RVD	RVD	RR (95% CI)	*P* Value^b^
		No. (%) GMC [Range]^a^	No. (%) GMC [Range]^a^		
All (N = 564)	Yes	107 (84.9)	19 (15.1)	0.52 (.34–.81)	.002
		108.5 [20.1–7297]^c^	57.2 [22.4–1017]^c^		
	No	311 (71.0)	127 (29.0)		
		<20	<20		
Vaccinated (n = 279)	Yes	69 (90.8)	7 (9.2)	0.47 (.22–1.00)	.037
		111.1 [20.1–7297]	77.6 [23.4–1017]		
	No	163 (80.3)	40 (19.7)		
		<20	<20		
Unvaccinated (n = 285)	Yes	38 (76.0)	12 (24.0)	0.65 (.39–1.09)	.079
		104.1 [20.9–3829]^d^	47.9 [22.4–338]^d^		
	No	148 (63.0)	87 (37.0)		
		<20	<20		

Abbreviations: CI, confidence interval; GMC, geometric mean concentration; RR, relative risk; RVD, rotavirus diarrhea.

^a^Comparison of GMC across groups performed by Mann-Whitney *U* test.

^b^ *P* value by χ^2^ or Fisher exact test.

^c^ *P* = .054.

^d^ *P* = .046.

Week 18 RV-IgG concentration was not associated with RVD (data not shown). In some infants, decline in RV-IgG from week 6 to week 18 was attenuated, suggesting replacement of waning maternal antibodies with RV-IgG induced by vaccination or infection. Infants with >2-fold decline in RV-IgG were more likely to experience RVD compared to those with ≤2-fold decline (OR, 2.7 [95% CI, 1.0–7.1]; *P* = .047). However, this was also an imprecise predictor of protection, as 12% of infants with ≤2-fold decline in RV-IgG still experienced RVD.

### RV-IgA Explains Little of Vaccine Effect According to Prentice Criteria

Prentice criteria [10] were used to evaluate RV-IgA as a surrogate marker for protection from RVD between weeks 18 and 52. Several conditions must be fulfilled for a marker to be considered a surrogate endpoint by Prentice criteria. The intervention must significantly affect the clinical and the surrogate outcomes, which Rotarix does. The surrogate must correlate with the clinical outcome; our results satisfy this condition. Finally, the surrogate must fully capture the vaccine group effect. As this condition is rarely fulfilled, methods to estimate the proportion of vaccine effect explained have been developed [17]. When evaluated by these methods, RV-IgA seropositivity explained 7.8% and RV-IgA concentration explained 13.2% of the vaccine effect (Table 4).

**Table 4.** Percentage of Rotarix Vaccine Effect Explained by Rotavirus-Specific Immunoglobulin A on Rotavirus Diarrhea, Weeks 18–52

Logistic Model	Variable	OR (95% CI)	*P* Value	% Vaccine Effect Explained by:
Vaccine arm +	Vaccine	2.72 (1.79–4.14)	<.001	Seropositivity: 7.8%
Seropositivity	Seropositivity	2.24 (1.31–3.81)	.003	
Vaccine arm +	Vaccine	2.57 (1.69–3.19)	<.001	RV-IgA (U/mL): 13.2%
Ln(RV-IgA)	Ln(RV-IgA)	0.70 (.56–.87)	.001	

Covariates included in each model: week 18 serum zinc concentration, exclusive breastfeeding at week 18, and treated water.

Abbreviations: CI, confidence interval; OR, odds ratio; RV-IgA, rotavirus-specific plasma IgA.

## DISCUSSION

In infants from an urban slum of Dhaka, Bangladesh, we detected weak population-level RV-IgA responses following vaccination and determined that RV-IgA was a suboptimal CoP for RV. Twenty-seven percent of infants seroconverted and 32% were seropositive postvaccination, lower frequencies than previously reported from the region [19–22]. GMC among vaccine recipients was 21 U/mL, compared with 13 U/mL among unvaccinated children, a clinically insignificant difference considering that postvaccination GMC is >200 U/mL in low-child-mortality countries and that durable protection may require GMC >90 U/mL [6]. This population demonstrated qualitative and quantitative defects in RV-IgA response: Few children responded, and those who did had relatively weak responses that were often short-lived. Bangladesh applied for Gavi approval in 2016, but Rotarix is not yet publicly available. Under current conditions, clinical effectiveness may fail to reach full potential.

The most important factor associated with impaired RV-IgA response in this study was maternally derived RV-IgG, supporting previous findings [23]. All vaccinated infants in the top decile for week 6 RV-IgG failed to seroconvert, suggesting that maternal antibodies must wane below a specific threshold for successful vaccination. Our study utilized delayed dosing; if these infants received Rotarix on the EPI schedule at 6 and 10 weeks, RV-IgA responses may have been further diminished. Most studies using delayed dosing vaccinated before 16 weeks, yielding modest results [20, 24]. Delaying vaccination further may be necessary to improve immunogenicity [24]. Further efforts to identify the optimum timing of vaccination are warranted; our data suggest initiating vaccination no earlier than week 10 and completing vaccination after week 17 as a possible starting point. This approach must be balanced against the challenge of altering established immunization schedules, and may be country-specific due to regional differences in age of peak RVD incidence [25]. Vaccination must be initiated early enough to induce protection prior to peak risk. In some regions, this approach may still fail: in Vellore, India, Rotarix dosing through 22 weeks of life did not improve seroconversion [26]. Increased age at vaccination may increase risk for intussusception [27]. However, as the number of deaths likely prevented by delayed immunization far exceeds intussusception risk, the World Health Organization has removed age restrictions for RV vaccines in LICs [28]; we believe that concern for intussusception should not hinder efforts to optimize dosing schedules.

A unique strength of this study is that we could evaluate RV-IgA and RVD risk at the individual level. Among seroconverters, risk was reduced but not eliminated, as 15% still experienced RVD by 1 year even at RV-IgA concentrations >1000 U/mL. RV-IgA in this population would underestimate protection, as VE in the parent cohort was 51% [12]. These findings support previous observations that population-level associations between RV-IgA and VE weaken in LICs [8]. In India, RV-IgA did not correlate with protection from RVD, and a threshold concentration that defined protection could not be identified [29]. Reanalysis of 2 seminal birth cohort studies from Mexico and India demonstrated that RV-IgA was not associated with RVD risk after controlling for age [30]. Surprisingly, 15% of vaccinated infants seroreverted between weeks 18 and 24, suggesting rapidly waning immunity in some children, which may help explain the discrepancy between postvaccination RV-IgA seroconversion and risk of future RVD.

For VE trials, the Prentice method is frequently employed to define the relationship between a surrogate endpoint and clinical outcome [8, 10]. According to Prentice criteria, RV-IgA only explained up to 13.2% of vaccine effect. In comparison, analysis of a Rotarix trial in South Africa and Malawi found that seropositivity explained 43.6% of vaccine effect against any RVD [9], and meta-analysis of 8 trials demonstrated a significant association between clinical VE and VE as predicted by RV-IgA [9]. The authors concluded that RV-IgA may be a useful correlate of VE for Rotarix trials. We also detected associations between RV-IgA and RVD, but the discordance between RV-IgA and population-level VE, RVD at the level of the individual child, and the scant proportion of vaccine effect explained suggests that RV-IgA is a poor CoP for RV in this setting at both the population and individual level. Our results underscore that RV-IgA is a nonmechanistic measure of immunogenicity that fails to capture other biologically relevant effects. For example, serum zinc is associated with protection from RVD [12] but was not associated with RV-IgA, suggesting mechanistic pathways for RV immunity that RV-IgA fails to reflect. Further efforts to identify alternate CoPs are therefore needed [8].

Other factors may contribute to reduced oral RV vaccine immunogenicity. Interference from oral poliovirus vaccine (OPV) is well documented and is greatest when the first doses of Rotarix and OPV are given concomitantly (typically at week 6), when OPV replication is highest [31]. In PROVIDE, infants received the first dose of Rotarix at week 10 with the second OPV dose, reducing potential interference, an added benefit of delayed dosing. Interference from nonpolio enteroviruses has also been described in this population [32] but is unlikely to be an easily modifiable risk factor. Levels of blood and stool biomarkers suggesting systemic and gut inflammation have been associated with Rotarix vaccine performance [13], but we were unable to model all variables because few infants had complete biomarker results. Breastfeeding was associated with impaired immunogenicity; others have demonstrated impacts of breast milk on vaccine infectivity and immunogenicity, but interventions addressing these factors (ie, withholding breastfeeding) have been unsuccessful [33]. Given its important health benefits, breastfeeding is an unattractive target for future interventions.

There are several limitations to this work. The EIA used in this study potentially underestimated RV-IgA concentration, likely due to antigen mismatch [34]. However, as 87.5% of tested specimens were concordant for serostatus when measured by both PROVIDE and Cincinnati EIA, the effect on seroconversion or seropositivity was likely minimal. We could not account for lack of natural RV exposure, which could help explain the discordance between lack of seroconversion but protection from RVD. However, Dhaka has among the highest rates of RV infection in the world [35], so the force of infection in this study was high. Neutralizing antibodies may have contributed but, due to limited specimen volume, could not be measured. The timing of antibody measurement likely impacted results. Asymptomatic infections after week 18 might increase RV-IgA, but this effect would be missed. However, week 24 RV-IgA results suggest that this was not a significant factor. Plasma collection at week 18, 1 week following the second Rotarix dose, may have been too early to reflect its full effect. The reason for blood draw at this time point was due to logistical constraints regarding frequency and timing of blood draws due to overall study design. Our results more accurately describe RV-IgA following the first dose. In phase 2 trials in high- and middle-income countries, seroconversion following 1 dose was 38%–88% [36–40], much higher than in our population. RV-IgA concentration was likely unaffected, since the major role of the second dose is for “catch-up” response among infants who did not initially respond, rather than boost antibody levels [36, 39, 40]. Although sample size was limited, this is supported by week 24 results among vaccinated infants. Seroconversion was modestly increased, but no differences were seen in RV-IgA concentration. Seroconversion at week 24 was still well below the 87% rate observed in high-income countries [6].

In summary, we have demonstrated poor RV-IgA responses in a birth cohort from urban Dhaka, Bangladesh. Increased maternally derived RV-IgG significantly decreased Rotarix vaccine immunogenicity and remains a potential target for population-level intervention. RV-IgA is a suboptimal CoP for RV in the developing world. Vaccine recommendations in regions with poor population-level RV-IgA responses must consider this limitation and should be based on efficacy data whenever possible. Further efforts to identify a reliable CoP for RV in LICs are needed to assist in future endeavors to improve vaccination strategies and evaluate next-generation vaccines.

## Supplementary Data

Supplementary materials are available at *Clinical Infectious Diseases* online. Consisting of data provided by the authors to benefit the reader, the posted materials are not copyedited and are the sole responsibility of the authors, so questions or comments should be addressed to the corresponding author.

Supplementary Materials

## Notes

***Acknowledgments.*** We thank the Performance of Oral Vaccines in Developing Countries study families and study staff for making this work possible.

***Financial support.*** This work was supported by the Bill & Melinda Gates Foundation (award numbers OPP1017093 to W. A. P. and OPP1127782 to B. D. K.); the International Centre for Diarrhoeal Disease Research, Bangladesh; and the governments of Bangladesh, Canada, Sweden, and the United Kingdom (core/unrestricted support).

***Potential conflicts of interest.*** M. M. M. reports laboratory services agreements with PATH, Merck, and GlaxoSmithKline, none of which were applicable to the work presented in this manuscript. B.L., M.C., D.M.D., E.R.C., and S.A.D. report funding from the Bill & Melinda Gates Foundation under the award listed for B.D.K. M.U.D., S.I., M.H.M., T.A.R., T.R.B., M.A., U.N., J.C.M., and F.Q. report funding from the Bill & Melinda Gates Foundation under the award listed for W.A.P. All other authors report no potential conflicts of interest. All authors have submitted the ICMJE Form for Disclosure of Potential Conflicts of Interest. Conflicts that the editors consider relevant to the content of the manuscript have been disclosed.
