# Supplementary material for: Rotavirus-Specific Immunoglobulin A Responses Are Impaired and Serve as a Suboptimal Correlate of Protection Among Infants in Bangladesh
Source: Clin Infect Dis. 2018 Jan 31;67(2):186–92. doi: 10.1093/cid/ciy076 (PMC6030840; doi:10.1093/cid/ciy076)
Supplement: Supplementary Materials [file ciy076_suppl_supplementary_materials.docx]

**Supplementary Materials**

Serial dilutions of each plasma specimen were measured. Optical density was measured and end-point titer calculated using the Thermo Multiskan system with Ascent software (ThermoScientific, Waltham, MA). A full titration of a RV antibody-positive control sample was performed in each assay plate to qualify each assay run.  A sample of this positive control was sent to Cincinnati to determine its concentration (U/mL) of both RV-IgA and RV-IgG.  The undiluted sample was determined to be 4000 U/mL for RV-IgA and 8000 U/mL for RV-IgG. Using this information, a conversion from endpoint titer to U/mL was performed using the following formula:  U/mL = A*B/C*4000/C, where A = Normalized titer for each unknown sample, B = normalized control titer, C = geometric mean of all normalized control titers, and 4000 = the conversion factor for U/mL, as determined by Cincinnati.  A similar formula was used for IgG using the constant of 8000 as described above.

| **Supplementary Table 1. Comparison of RV-IgA measurements by CCHMC and iccdr,b EIA** | | | | | |
| --- | --- | --- | --- | --- | --- |
| Week 18 RV-IgA (U/mL) | |  | Seropositive (yes/no) | | |
| CCHMC | icddr,b |  | CCHMC | icddr,b | Concordant? |
| <7.50 | <7.50 |  | No | No | Yes |
| <7.50 | <7.50 |  | No | No | Yes |
| <7.50 | <7.50 |  | No | No | Yes |
| <7.50 | <7.50 |  | No | No | Yes |
| <7.50 | 24.21 |  | No | Yes | No |
| <7.50 | 17.27 |  | No | No | Yes |
| <7.50 | <7.50 |  | No | No | Yes |
| <7.50 | <7.50 |  | No | No | Yes |
| <7.50 | <7.50 |  | No | No | Yes |
| <7.50 | <7.50 |  | No | No | Yes |
| <7.50 | <7.50 |  | No | No | Yes |
| <7.50 | <7.50 |  | No | No | Yes |
| <7.50 | <7.50 |  | No | No | Yes |
| <7.50 | <7.50 |  | No | No | Yes |
| <7.50 | <7.50 |  | No | No | Yes |
| <7.50 | <7.50 |  | No | No | Yes |
| <7.50 | <7.50 |  | No | No | Yes |
| <7.50 | <7.50 |  | No | No | Yes |
| <7.50 | <7.50 |  | No | No | Yes |
| <7.50 | 10.59 |  | No | No | Yes |
| <7.50 | 8.67 |  | No | No | Yes |
| <7.50 | 4.44 |  | No | No | Yes |
| <7.50 | 6.74 |  | No | No | Yes |
| <7.50 | 1.96 |  | No | No | Yes |
| <7.50 | 0.09 |  | No | No | Yes |
| <7.50 | -0.03 |  | No | No | Yes |
| 8.93 | 1.47 |  | No | No | Yes |
| 9.68 | 5.25 |  | No | No | Yes |
| 13.58 | 11.37 |  | No | No | Yes |
| 19.88 | 42.62 |  | No | Yes | No |
| 41.78 | 14.47 |  | Yes | No | No |
| 51.00 | 3.52 |  | Yes | No | No |
| 95.40 | 22.21 |  | Yes | Yes | Yes |
| 107.42 | 12.94 |  | Yes | No | No |
| 374.51 | 144 |  | Yes | Yes | Yes |
| 471.00 | 1361.95 |  | Yes | Yes | Yes |
| 666.81 | 303.28 |  | Yes | Yes | Yes |
| 1132.78 | 30.96 |  | Yes | Yes | Yes |
| 4321.41 | 70.9 |  | Yes | Yes | Yes |
| 19463.69 | 7297.39 |  | Yes | Yes | Yes |
| Abbreviations: CCHMC, Cincinnati Children’s Hospital Medical Center; EIA, enzyme immunoassay; icddr,b, International Centre for Diarrhoeal Disease Research, Bangladesh; RV-IgA, plasma rotavirus-specific immunoglobulin A. | | | | | |
